# Natural and enriched Cr target development for production of Manganese-52

**DOI:** 10.1038/s41598-022-27257-w

**Published:** 2023-01-20

**Authors:** Jennifer M. Pyles, James M. Omweri, Suzanne E. Lapi

**Affiliations:** 1 Department of Radiology, University of Alabama at Birmingham, Birmingham, AL USA; 2 Department of Chemistry, University of Alabama at Birmingham, Birmingham, Al USA

**Keywords:** Chemistry, Inorganic chemistry, Nuclear chemistry

## Abstract

^52^Mn is a promising PET radiometal with a half-life of 5.6 days and an average positron energy of 242 keV. Typically, chromium of natural isotope abundance is used as a target material to produce this isotope through the ^nat/52^Cr(p,n)^52^Mn reaction. While natural Cr is a suitable target material, higher purity ^52^Mn could be produced by transitioning to enriched ^52^Cr targets to prevent the co-production of long-lived ^54^Mn (t_1/2_ = 312 day). Unfortunately, ^52^Cr targets are not cost-effective without recycling processes in place, therefore, this work aims to explore routes to prepare Cr targets that could be recycled. Natural Cr foils, metal powder pellets, enriched chromium-52 oxide and Cr(III) electroplated targets were investigated in this work. Each of these cyclotron targets were irradiated, and the produced ^52^Mn was purified, when possible, using a semi-automated system. An improved purification by solid-phase anion exchange from ethanol-HCl mixtures resulted in recoveries of 94.5 ± 2.2% of ^52^Mn. The most promising target configuration to produce a recyclable target was electroplated Cr(III). This work presents several pathways to optimize enriched Cr targets for the production of high purity ^52^Mn.

## Introduction

Manganese-52 (^52^Mn) is a positron emitting radiometal which can be used for long-lived studies using positron emission tomography (PET). Additionally, since non-radioactive Mn has previously been used as a contrast enhancing agent for Manganese Enhanced MRI (MEMRI), radioactive ^52^Mn can be combined with the nonradioactive Mn to create a dual modality signal enhancing contrast agent for imaging with PET/MRI^1^.

One of the most common routes of production for ^52^Mn is ^nat/52^Cr(p,n)^52^Mn. Several researchers have reported on the preparation and characterization of Cr targets of natural isotopic abundance (natural Cr). (Table 1). Other methods have used Vanadium targets to make ^52^Mn via bombardment with ^3^He, however, there are only a limited number of accelerators that have the capabilities to accelerate these particles^2^.

**Table 1:** Prior natural Cr and V target configurations.

Author	Year	Target	Preparation method	Target backing	Nuclear reaction	Products
Topping et al*.*^3^	2013	Natural Cr	Foil	Al	(p,n) (p,n) (p,pn) (p,n)	^52^Mn ^52m^Mn ^51^Cr ^54^Mn
Graves et al*.*^4^	2015	Natural Cr	Pressed powder using a hydraulic press	Ag	(p,n) (p,n) (p,pn) (p,n)	^52^Mn ^52m^Mn ^51^Cr ^54^Mn
Fonslet et al.^5^	2016	Natural Cr	Pressed powder using a mechanical press	Ag	(p,n) (p,n) (p,pn) (p,n)	^52^Mn ^52m^Mn ^51^Cr ^54^Mn
Saar et al*.*^2^	2018	Natural V	Pressed pellets	Al cup	(He-3,2n)	^52^Mn
Bianchi et al*.*^6^	2020	Natural Cr	Electro-deposition	Al/Zn substrate	(d,xn) (d,xn) (d,pxn) (d, xn) (d,x)	^52^Mn ^52m^Mn ^51^Cr ^54^Mn ^48^V
Brezovesik et al*.*^7^	2020	Natural Cr	Pressed pills	N/A	(p,n) (p,n) (p,pn) (p,n)	^52^Mn ^52m^Mn ^51^Cr ^54^Mn
Sciacca et al*.*^8^	2021	Natural Cr	Spark plasma sintering (SPS)	(Au/Cu)	(p,n) (p,n) (p,pn) (p,n)	^52^Mn ^52m^Mn ^51^Cr ^54^Mn

Natural Cr consists of 4 isotopes of Cr including ^50^Cr (4.35%), ^52^Cr (83.79%), ^53^Cr (9.50%), and ^54^Cr (2.37%). Since natural Cr contains a high percentage of ^52^Cr, it is an inexpensive route to produce ^52^Mn. Unfortunately, the reactions that occur on the other three stable isotopes of Cr can lead to the production of several radiocontaminants via this irradiated target material as shown in Table 2. If the cross sections and thresholds of these reactions are taken into consideration and the proton beam energy is kept below ~ 13 MeV, only two of these impurities, ^52m^Mn and ^54^Mn, are observed in the gamma spectra after irradiation of the natural chromium^1,9–11^. Previous studies have optimized the bombardment parameters through cross section measurements^10^. Another solution to prevent the production of these long lived impurities would be to use an enriched ^52^Cr target.

**Table 2:** Potential products and impurities through the reactions ^nat^Cr(p,x)X.

Target material (abundance)	Nuclear reaction	Half-life	Threshold (MeV)	Daughter
^50^Cr (4.3%)	^50^Cr(p, n)^50^Mn ^50^Cr(p, pn)^49^Cr ^50^Cr(p, α)^47^ V	1.74 min 42.3 min 32.6 min	8.82 13.26 26.25	^50^Cr (stable) ^49^V (330 days) ^47^Ti (stable)
^53^Cr (9.5%)	^53^Cr(p, n)^53^Mn	3.7 × 10^6^ yr	5.63	^53^Cr (stable)
^52^Cr (83.8%)	^**52**^**Cr(p, n)**^**52**^**Mn** ^**52**^**Cr(p, n)**^**52m**^**Mn** ^52^Cr(p, pn)^51^Cr ^52^Cr(p,2n)^51^Mn ^52^Cr(p, α)^49^ V	5.59 days 21.10 min 27.70 days 46.2 min 331.00 days	5.66 5.66 13.26 16.34 26.25	^52^Cr (stable) ^52^Cr (stable) ^51^V (stable) ^51^Cr (27.7 days) ^49^Ti (stable)
^54^Cr (2.4%)	^**54**^**Cr(p, n)**^**54**^**Mn**	312.20 days	5.59	^54^Cr (stable)

The bolded reactions are the most common products observed. This work used a maximum irradiation energy of 12.9 MeV while the cross section from El Sayed et al., 2019 at that energy is 249.8 mb. (Modified from El Sayed et al. 2019)^10,12^.

Therefore, we aimed to develop enriched ^52^Cr targets using chemistry which would enable recycling of the target material to make new targets at a reduced cost. Natural Cr targets were used to carry out feasibility studies for production, purification, and recycling processes before the transition to ^52^Cr. Cr can be purchased in many different forms including powder, foils, rods, however, ^52^Cr is typically obtained in metal powder form. Cr powders can be pressed into powder targets using a hydraulic press, reacted to produce other chemical forms, such as, Cr_2_O_3_ or CrCl_3_, or electroplated to increase the potential for recyclable enriched ^52^Cr targets. Since Cr(VI) is considered to have higher toxicity this work avoided that oxidation state and focused on the less toxic Cr(III). This work examined various species and target configurations of Cr to explore recyclable enriched ^52^Cr targets.

## Materials and methods

### Target designs

#### General materials

Natural Cr metal powder with 5N purity, Ta sheets with 3N8 purity, Al sheets with 5N purity, Cu sheets with 5N5 purity, Au sheets with 5N purity and Pt rods with 3N5 purity were obtained from ESPI Metals, Ashland, OR. Enriched ^52^Cr metal powder with 98.8% enrichment was obtained from Isoflex, San Francisco, CA. Viton O-rings were obtained from McMaster-Carr, Elmhurst, IL. The 10 mm diameter ID dry pressing die set was obtained from Across International (Livingston, NJ). Chromium chloride hexahydrate with purity ≥ 98% and 1 mL SPE tubes with frits were obtained from Millipore Sigma, Burlington, MA. AG1-X8 resin analytical grade 100–200 mesh chloride form was obtained from Bio-Rad, Hercules, CA. A mixed nuclide source in a sealed 1.5 mL centrifuge tube used for high purity germanium detector (HPGe) calibration was prepared by Eckert & Ziegler Analytics (Atlanta, GA). 1,4,7,10-tetraazacyclododecane-1,4,7,10-tetraacetic acid (DOTA) was obtained from Macrocyclics (Plano, TX) and the Aluminum backed Si-60 TLC plates were obtained from Sorbtech technologies (Norcross, GA).

All other materials were purchased from Fisher Scientific (Hampton, NH) unless stated otherwise.

#### Natural Cr foils

As reported in Pyles et al. 2021, Cr foils were produced on a large scale by electrodeposition of natural abundance Cr as previously described in Wooten et al. 2015^11,13^.

#### Natural Cr powder targets

**Figure 1:**
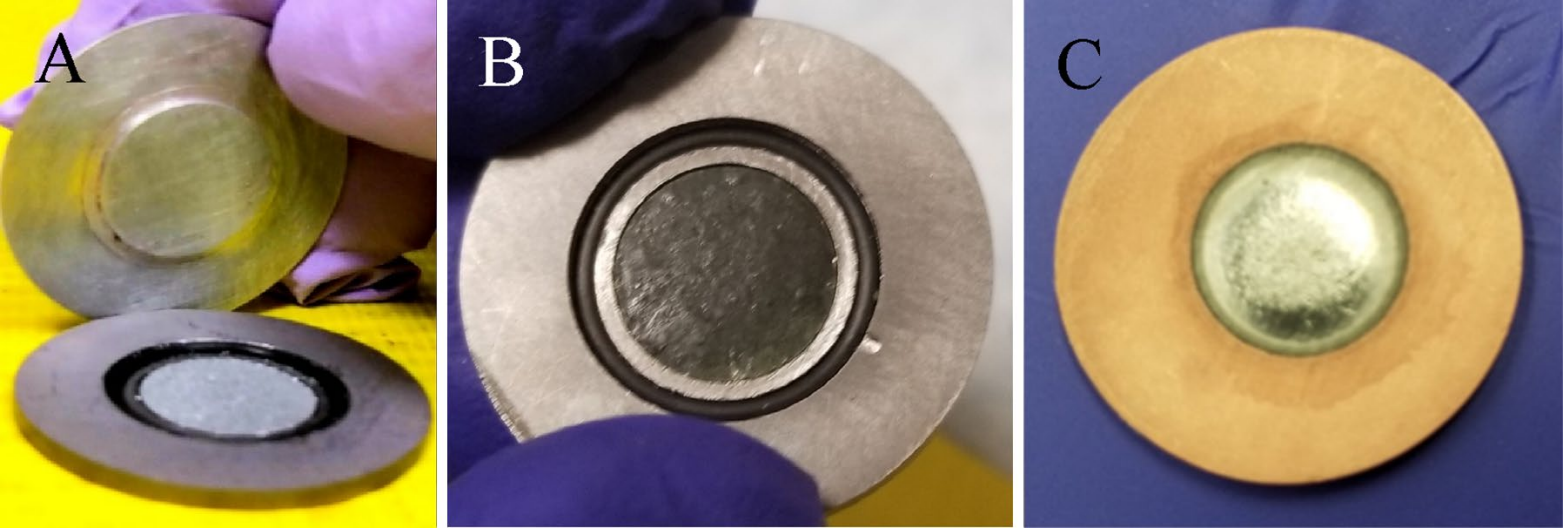
Photos of target configurations described before irradiation. (**A**) Cr metal powder target with Al “push button” degrader, (**B**) Cr_2_O_3_ target, (**C**) electroplated Cr(III) target.

Cr metal powder targets were made by pressing 200–230 mg of natural Cr metal powder at 5 tons for 5 min using a hydraulic press and 10 mm dye set. These targets were 10 mm in diameter and had 0.4–0.5 mm nominal thickness. Tantalum (Ta) sheets with 3N8 purity and thickness of 0.06 in, were cut into a 2.5 cm diameter coins and a 0.5 mm deep divot was machined into the center to hold the Cr powder target. Additionally, these targets had a divot for a Viton O-ring outside of the target divot as to prevent the loss of target material. The targets were finally capped with a 0.75 mm Aluminum (Al) degrader with a “push button” design to fit into the 14 mm divot to cover both the target and the O-ring as shown in Fig. 1A.

#### Enriched Chromium oxide powder targets

^52^Cr_2_O_3_ was made by the reactions.

$$^{{{52}}} {\text{Cr }} + {\text{ 3HCl}} \to^{52} {\text{CrCl}}_{{3}} + {\text{ 3H}}^{ + } {; }^{52} {\text{CrCl}}_{{3}} + {\text{ 3NH}}_{{4}} {\text{OH}} \to^{{{52}}} {\text{Cr}}\left( {{\text{OH}}} \right)_{{3}} + {\text{ 3NH}}_{{4}} {\text{Cl }};{ 2}^{{{52}}} {\text{Cr}}\left( {{\text{OH}}} \right)_{{3}} \to^{{{52}}} {\text{Cr}}_{{2}} {\text{O}}_{{3}} + {\text{ 3H}}_{{2}} {\text{O}}$$

Using enriched ^52^Cr metal powder as a starting material.

200–230 mg of ^52^Cr was dissolved in 5 mL of 6 M hydrochloric acid (HCl) heated to 95 °C for approximately 30 min or until dissolved to yield ^52^CrCl_3_. Approximately 10 mL of 7 M ammonium hydroxide was added to the ^52^CrCl_3_ solution to precipitate the material as ^52^Cr(OH)_3_. The ^52^Cr(OH)_3_ was then centrifuged at 3000 rpm for 7 min or until the precipitate was separated from the solution. The precipitate pellet was separated from the supernatant, and rinsed with MQ water. The ^52^Cr(OH)_3_ was heated at 200–250 °C to obtain the final product of ^52^Cr_2_O_3_. Then the ^52^Cr_2_O_3_ powder was weighed and placed in the oven to keep it dry at 250 °C. The powder targets were made by pressing 200–230 mg of ^52^Cr_2_O_3_ powder in a 10 mm diameter ID dry pressing die set, to ensure the correct target shape, at 5 tons for 5 min using a hydraulic press to make the pressed pellet used for irradiations. These targets were 10 mm in diameter and had 0.4–0.5 mm nominal thickness. The target was configured as described in Section 1.3 with the Ta backing and Al “push button” degrader as shown in Fig. 1B.

#### Electroplated Cr(III) targets

**Figure 2:**
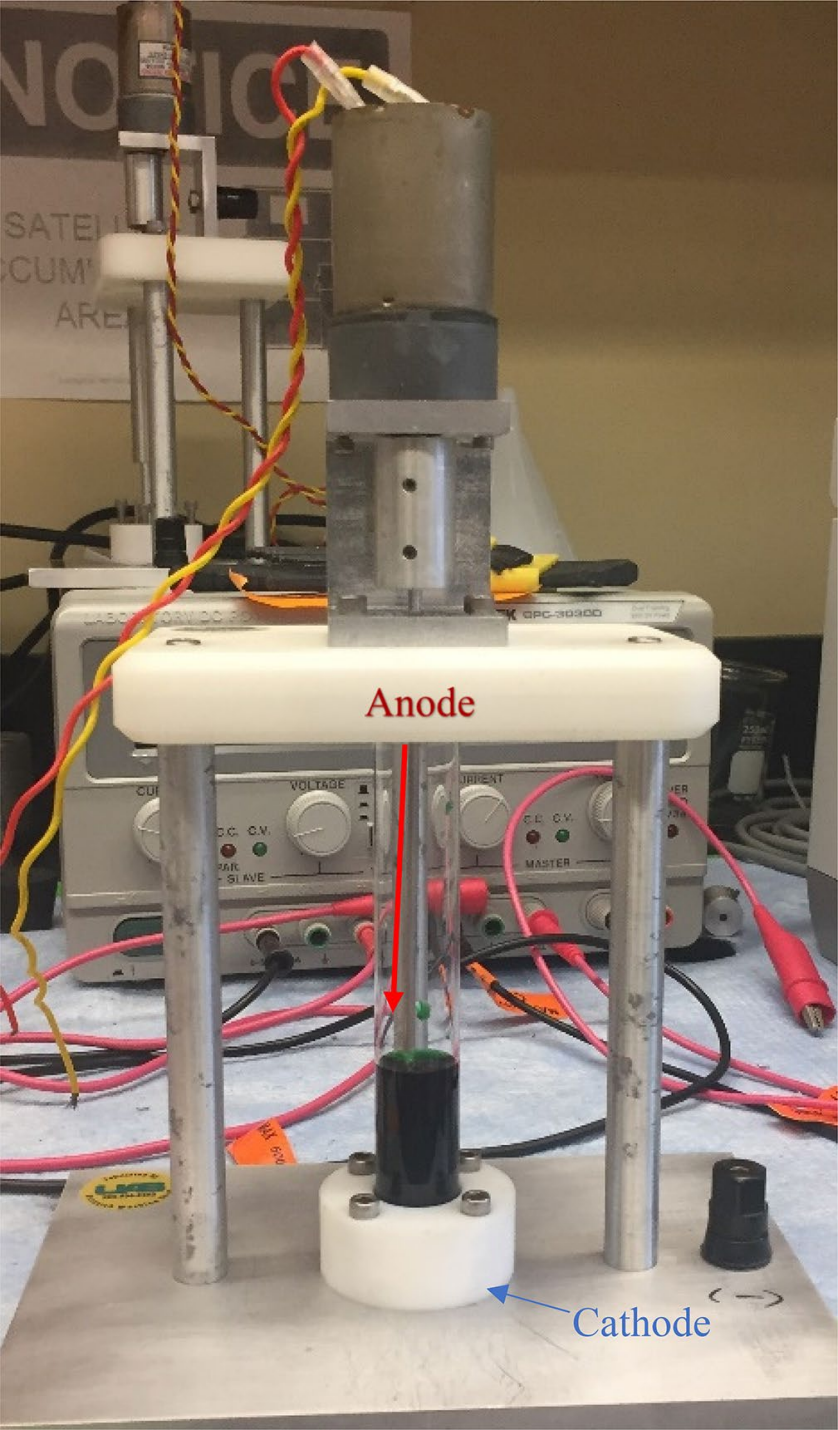
Electroplating apparatus used to plate Cr metal from a CrCl_3_ solution where the cathode is located between the base plate and the plastic coin holder. The anode is the platinum rod which can be seen connected to the motor and suspended in the CrCl_3_ plating solution.

Chromium chloride hexahydrate (CrCl_3_·6H_2_O) was utilized as the chromium source in the electrodeposition solution which was adapted from the procedure previously described in Liang et al.^14^. A 20 × 150 mm lime glass disposable culture tube modified to have two open ends was used as the electrolysis cell. The plating diameter was 10 mm with a 0.1–0.5 mm nominal thickness depending on the amount of Cr plated as shown in Fig. 1C. Copper (Cu) or Gold (Au) sheets with 5N5 and 5N purity, respectively, and thickness of 0.75 mm were cut into a 2.5 cm diameter coins to be used as the target backing (cathode) while a platinum rod was used as the rotating anode suspended in the solution. The electroplating apparatus was connected to a DC power supply that utilized alligator clips in order to apply voltage to the platinum rod. (Fig. 2) The voltage applied to the platinum rod and the electroplating solution was 3.8 V which supplied a current averaging 0.075 A. Additionally, these targets were finally capped with a 0.75 mm Al degrader.

### Irradiation parameters

The stopping and range of ions in matter (SRIM) was used to determine the energy of the proton beam on target after the degrader^15^. All bombardments were performed on the TR-24 Cyclotron (Advanced Cyclotron Systems Inc). The irradiations below used bombardment parameters previously optimized by El Sayed et al. These target configurations were irradiated with an incident proton beam energy of 17.5 MeV on the degrader (12.8 MeV on the Cr target material) at 15 μA for 2–8 h. The proton beam was stopped in the backing coin described in each target configuration. The target was cooled by He gas on the front of the target while the back of the target was water cooled.

### Target characterization via scanning electron microscope (SEM)

The electroplated targets were analyzed by the SEM to determine the purity of the plated Cr. SEM analysis was performed with a SEM FEI Quanta 650 FEG with secondary electron detector at an acceleration voltage of 16 kV for spectroscopy equipped with an electron dispersive X-ray spectroscopy (EDAX) analyzer to measure qualitatively the sample stoichiometry. The SEM utilized the xT microscope control software while the EDAX used Teams software.

### Purification methods

The purification method described below was adapted from Pyles et al. except for the natural Cr foils which used the exact separation process written in Pyles et al.^13^. For the adapted studies, three columns composed of 1 mL solid-phase extraction (SPE) tubes were loaded with AG1-X8 resin as follows: column 1 (C1)—300 mg, column 2 (C2)—200 mg and column 3 (C3)—100 mg. A frit provided with the SPE tubes was added on top of the AG1-X8 resin to prevent the resin bed from being disturbed by the incoming reagents during the purification process. The irradiated Cr target was dissolved in concentrated HCl, diluted to 3% HCl in EtOH and loaded onto a column containing AG1-X8 resin. The Cr was eluted with 3% HCl in ethanol. The Mn was eluted with 6 M HCl.

**Figure 3:**
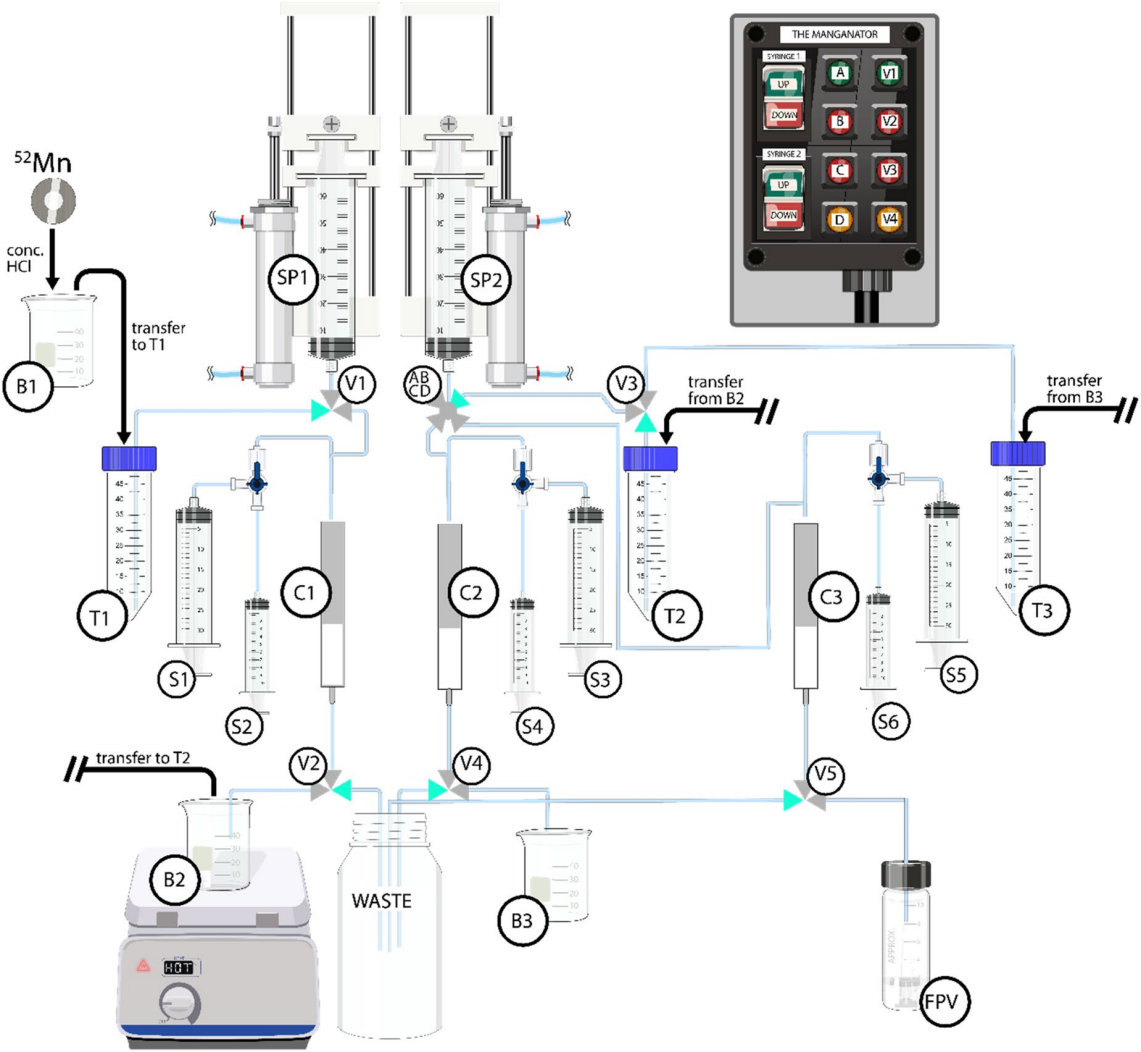
Adapted schematic of semi-automated system designed to separate ^52^Mn from the chromium target material, valve control and syringe pumps while syringes 1–6 are operated manually. B1-3: Beaker 1–3; T1-3: 50 mL conical vial 1–3; C1-3: Column 1–3; V1-5: Valve 1–5; ABCD: 4-way valve; S1-6: Syringe 1–6; SP1-2: Syringe pump 1–2; FPV: Final product vial. Modified from Pyles et al.^13^.

The adapted procedure of the three-column chemistry separation is described in Table 3 and Fig. 3.

**Table 3:** Chemical separation procedure for the load-wash-elute sequence for a three-column system for the purification of ^52^Mn from the cyclotron bombarded Cr targets^13^.

Step	Purpose	Column (pump)	Reagent	Volume (mL)	Valve position				
					V1	V2	V3	V4	V5
1	Load	C1 (SP1)	Cr/Mn crude 3% HCl in ethanol	50	NO	NC	NC	NC	NC
2	Wash	C1 (S1)	3% HCl in ethanol	25	NO	NC	NC	NC	NC
3	Elute	C1 (S2)	0.1 M HCl	5	NO	NO	NC	NC	NC
4	Load	C2 (SP2)	Mn in 3% HCl in ethanol	50	NC	NC	NO	NC	NO
5	Wash	C2 (S3)	3% HCl in ethanol	25	NC	NC	NO	NC	NC
6	Elute	C2 (S4)	0.1 M HCl	5	NC	NC	NC	NO	NC
7	Load	C3 (SP2)	Mn in 3% HCl in ethanol	50	NC	NC	NO	NC	NC
8	Wash	C3 (S5)	3% HCl in ethanol	25	NC	NC	NO	NC	NC
9	Elute	C3 (S6)	0.1 M HCl	5	NC	NC	NC	NC	NO

### Gamma spectroscopy

After irradiation and target dissolution, aliquots were collected from the crude solution of dissolved ^nat^Cr and ^52^Cr targets before and after purification. Samples were analyzed as described in Pyles et al.^13^.

### Inductively coupled plasma-mass spectrometry (ICP-MS)

Inductively Coupled Plasma-Mass Spectroscopy (ICP-MS, 7700 Series; Agilent, Santa Clara, CA) was used to evaluate the presence of Cr in the dissolved target material and the solutions eluted from each column. Aliquots of each sample were taken and analyzed as described in Pyles et al.^13^.

### Chelation chemistry

To assess the chemical purity and apparent molar activity (AMA) of the ^52^Mn produced by the natural chromium metal powder targets, a chelation assay was carried out as previously described by Graves et al.^4^. Briefly ^52^MnCl_2_ was dried down and reconstituted in 0.1 M ammonium acetate, pH 4.5. A twofold serially diluted samples of DOTA (1,4,7,10-tetraazacyclododecane-1,4,7,10-tetraacetic acid) 1 mg/mL in 0.1 M ammonium acetate, pH 4.5 were prepared. 100 μL of buffered activity was combined with each of the DOTA samples and incubated at 37 °C for 1 h. The labeling efficiency of the samples was assessed by spotting 2 μL of each sample on Aluminum backed Si-60 TLC plates. The plates were developed in 0.1 M HCl mobile phase, ^52^Mn-DOTA remained at the origin and free ^52^Mn moved with the mobile phase.

## Results and discussion

### Target preparation and yields

The details of each target configuration’s irradiation and purification results are listed in Table 4. The theoretical values for each target were calculated using the thick target yield measurement and are in agreement with the experimental yields.

**Table 4:** Target configuration and radioactivity before purification and purification yield results with variable currents and irradiation times.

Target/degrader	Al degrader	2 stacked Cr foils (n = 3)	Cr metal powder (n = 3)	^52^Cr_2_O_3_ (n = 2)	Electroplated Cr(III) (n = 3)
Weight (mg)	N/A	100–120	200–230	200–230	40–75
Thickness (mm)	0.75	0.25	0.4	0.5	0.24
Incident energy (MeV)	17.5	12.5	12.5	12.5	12.5
Exit energy (MeV)	12.5	7.5	1.5	6	7.6
Irradiation time (h)	N/A	6.5	8	2.5	2
Theoretical activity MBq (mCi)	N/A	284.9 (7.7)	355.2 (9.6)	321.9 (8.7)	129.5 (3.5)
Experimental activity MBq (mCi)	N/A	284.9 ± 48.1 (7.7 ± 1.3)	336.7 ± 44.4 (9.1 ± 1.2)	318.2 ± 7.4 (8.6 ± 0.2)	118.4 ± 7.4 (3.2 ± 0.2)
Percent recovery (%)	N/A	70.8 ± 3.3	93.1 ± 2.5	0	95.9 ± 1.8
Purification time (h)	N/A	8.2 ± 0.6	4.3 ± 0.5	0	4.1 ± 0.3

### Natural chromium foils

In the original target configuration of 1–2 natural Cr foil(s) and purification method previously described in Pyles et al., the foils are no longer available and two–three columns were used^13^. The amounts of Cr and nonradioactive Mn in the final product per batch [batch size: 284.9 ± 48.1 MBq (7.7 ± 1.3 µCi)] for the radioactive purifications were 5.6 ± 8.6 μg and 1.3 ± 2.3 μg, respectively. The iron and copper contaminants for the radioactive purifications per batch were 79 ± 14 µg (1.41 µmol) and 54 ± 2 µg (0.85 µmol), respectively.

### Natural chromium powder

This target configuration was determined to be the most successful in terms of ease of overall use. High yields of 336.7 ± 44.4 MBq (9.1 ± 1.2 mCi) were obtained after an 8 h irradiation. These targets were simple to assemble and purify with high percent recoveries of ^52^Mn at 93.1 ± 2.5%. The purification system separated Mn from the Cr target material by using a series of three 1 mL SPE tubes. The target purification for of natural chromium powder [batch size: 336.7 ± 44.4 MBq (9.1 ± 1.2 µCi)] resulted in ICP-MS data for Cr (10.5 ± 2.6 µg), Mn (2.0 ± 0.6 µg), Fe (7.2 ± 1.3 µg), Cu (2.9 ± 0.4 µg) and Zn (13.2 ± 1.0 µg) for each of the final products. One downfall is this target cannot be recycled and therefore was not deemed a good candidate to be converted to an enriched ^52^Cr target unless recycling was not necessary.

### Enriched chromium-52 oxide powder

Then the ^52^Cr_2_O_3_ powder was weighed and 411.5 ± 44.5 mg was placed in the oven to keep it dry at 250 °C. These target configurations were very similar to the natural chromium metal powder, however, the enriched ^52^Cr metal was used to make the ^52^Cr_2_O_3_. The enriched material resulted in higher yields in a shorter time of 318.2 ± 7.4 MBq (8.6 ± 0.2 mCi) in 2.5 h. Unfortunately, this material did not dissolve in any of the following acids and bases in their concentrated forms: hydrochloric acid, nitric acid, sulfuric acid, hydrofluoric acid, and sodium hydroxide. Mixtures of these acids were also attempted without success using aqua regia and piranha solution. This is in line with prior literature reports that harsh conditions are required to dissolve Cr_2_O_3,_ therefore, this target could not be purified to obtain the purified ^52^Mn^16,17^. If a reasonable dissolution was developed these targets could be recycled and reused for an enriched ^52^Cr target. The recycling process would utilize the reaction listed to make the ^52^Cr_2_O_3_ starting with the CrCl_3_ that is collected during the purification process.

### Electroplated chromium(III)

**Figure 4:**
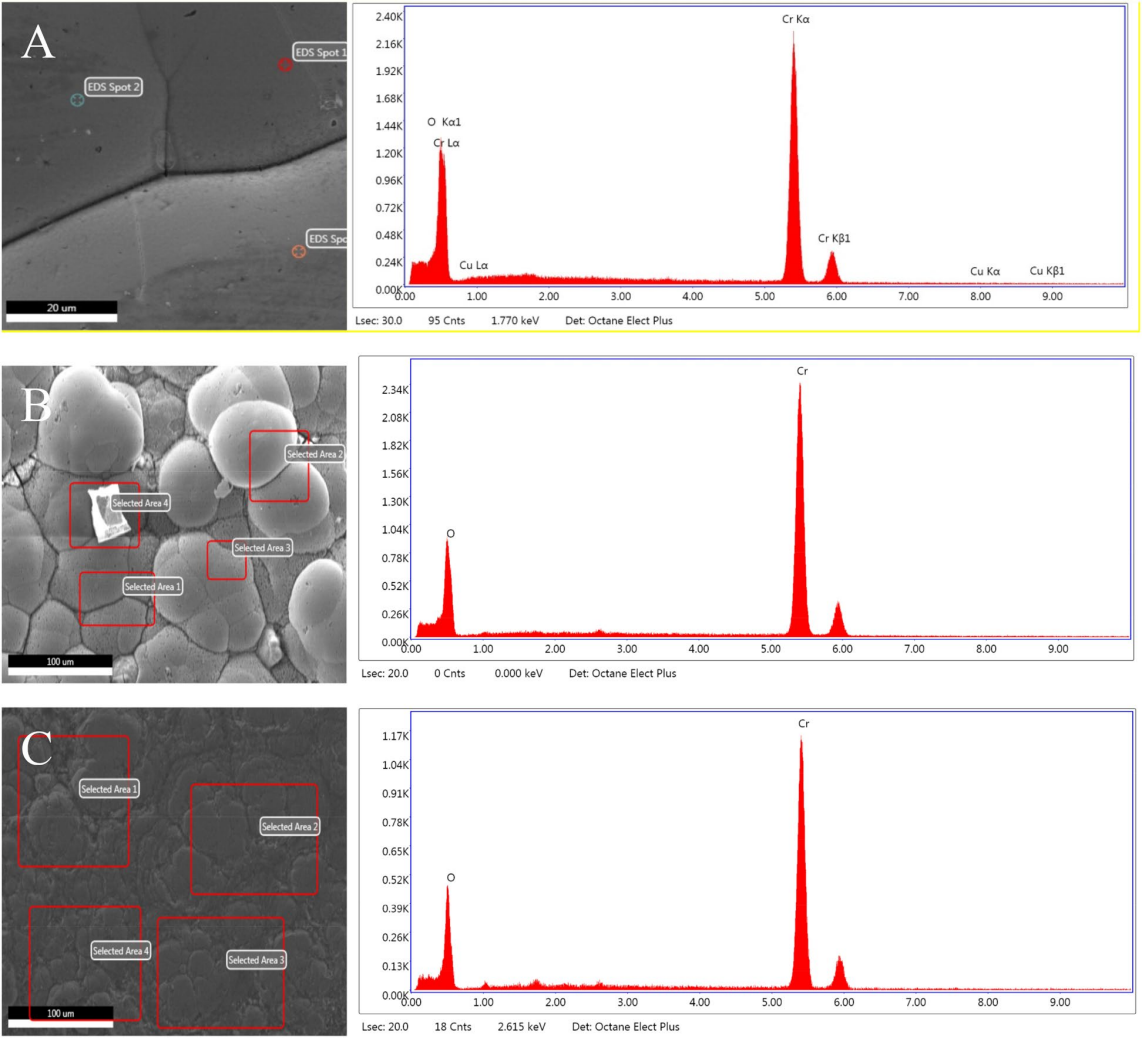
Scanning electron microscope (SEM) image and the energy dispersive X-ray analysis (EDAX) spectra for a representative electroplated Cr(III) target with a (**A**) Cu backing, (**B**) Au backing (**C**) Au backing after sanding.

The electroplated Cr utilizes chromium oxide hexahydrate in the electrodeposition solution. The formic acid and urea were used as complexing agents while the ammonium chloride and sodium chloride were used as conducting salts and finally the methanol and boric acid were used as buffering agents. These targets were further investigated by a scanning electron microscope (SEM) (Fig. 4). The analytical technique confirmed that the electroplated targets contained 95.6 ± 1.0% by weight percent and 89.3 ± 1.1% by atomic percent of Cr on the surface with 3.2 ± 0.2% by weight percent and 9.7 ± 0.6% by atomic percent of oxygen using a Cu backing. The electroplated targets using an Au backing contained 93.5 ± 1.5% by weight percent and 83.1 ± 3.9% by atomic percent of Cr on the surface with 5.6 ± 1.2% by weight percent and 16.5 ± 3.1% by atomic percent of oxygen before sanding the surface and contained 93.7 ± 0.6% by weight percent and 82.0 ± 1.4% by atomic percent of Cr on the surface with 6.3 ± 0.6% by weight percent and 18.0 ± 1.4% by atomic percent of oxygen after sanding the surface. While the amount of Cr is slightly increased on the Cu backing, the Au is more practical as elevated Cu in the final product was observed when the Cu backings were used due to slight dissolution of Cu during the dissolution of the Cr target material. Plating yields were 16.8 ± 2.6% which could be improved for conversion to an enriched recycled target.

**Figure 5:**
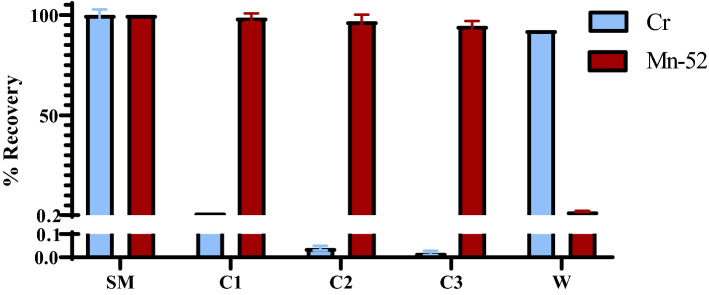
ICP-MS and HPGe data of radioactive purifications of ^52^Mn using the three-column semi-automated system. The results are expressed in Average ± SD; N = 3. *SM* Starting Material, *C1* Column 1, *C2* Column 2, *C3* Column 3, *W* Waste material.

The purification system separated Mn from the Cr target material by using a series of three 1 mL SPE tubes. The results from the radioactive purifications were obtained using the electroplated Cr(III) targets shown in Fig. 5. The target purifications for electroplated Cr(III) targets [batch size: 118.4 ± 7.4 MBq (3.2 ± 0.2 µCi)] resulted in ICP-MS data for Cr (1.0 ± 0.5 µg), Mn (0.6 ± 0.03 µg), Fe (4.9 ± 1.6 µg), Cu (4.5 ± 0.3 µg) and Zn (14.3 ± 2.3 µg) for each final product. Additionally, the improved purification process increased the percent yield of ^52^Mn from 70.8 ± 3.3% (n = 3) (Pyles et al.) to 94.5 ± 2.2% when both the electroplated and chromium metal powder targets are considered.

### Gamma spectroscopy

**Figure 6:**
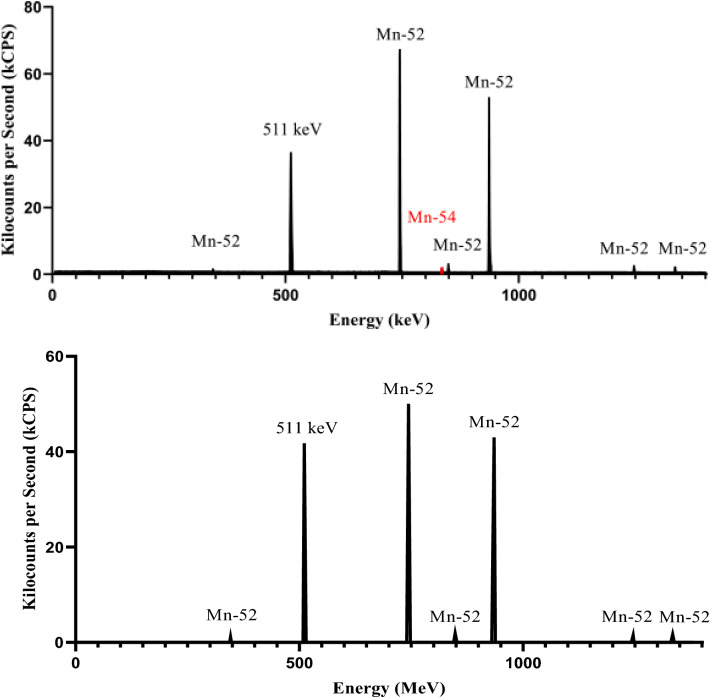
Top. Representative gamma spectra taken after the ^52^Mn purification process of the natural Cr metal powder target (all natural targets discussed showed similar spectra with the same peaks) showing a lack of ^51^Cr peak (320.1 keV). However, the ^54^Mn peak (835.85 keV), highlighted in red, can still be observed as well as the many characteristic peaks of ^52^Mn (345.8, 744.23, 847.7, 935.54, 1246.28, and 1333.65 keV) when natural Cr targets are used. Bottom. Representative gamma spectra taken after the ^52^Mn purification process showing the absence of the ^54^Mn peak when the enriched ^52^Cr metal powder target was used^1,13^.

The radionuclidic purity of the samples was verified via gamma-ray spectroscopy where samples were acquired before, during and after purification of ^52^Mn. ^52m^Mn (a meta-stable state of ^52^Mn with a t_1/2_ = 21.1 min) and ^50^Mn were not present in the spectra due to their short half-lives. ^51^Cr was not present in the spectra where the limit of detection was less than 1.24 ± 0.32%. The amount of ^54^Mn detected in the final sample was 0.125 ± 0.124% of the total sample displayed in the top gamma spectra of Fig. 6. When an enriched ^52^Cr target was used the ^54^Mn was less than the limit of detection of 0.01 ± 0.001% displayed in the bottom gamma spectra of Fig. 6.

### Chemical purity

**Figure 7:**
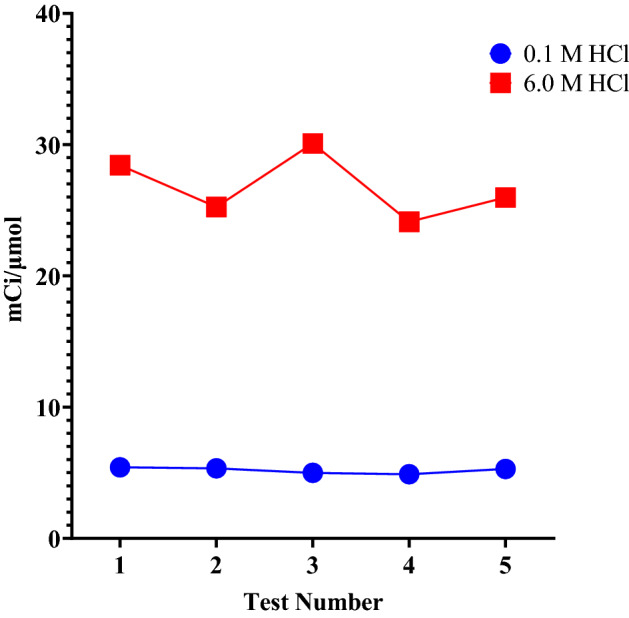
Apparent molar activity of ^52^Mn as a function of the concentration of the elution solution.

Varying labeling efficiencies of the macrocyclic chelator DOTA with ^52^Mn in 0.1 M ammonium acetate, pH 4.5 at 37 °C was observed utilizing the natural Cr metal powder targets. The apparent molar activity was found to depend on the concentration of the eluant, especially for the natural chromium powder targets. When ^52^Mn was eluted with 0.1 M HCl, the apparent molar activity (AMA) was 185 ± 22.2 MBq/µmol (5.0 ± 0.6 mCi/μmol) and when 6 M HCl was used, the AMA increased to 999 ± 88.8 MBq/µmol (27.0 ± 2.4 mCi/μmol) as shown in Fig. 7.

## Discussion

The Cr target configurations presented explored additional techniques for the possibility of an enriched ^52^Cr target that would improve the production of ^52^Mn. The novel targets have challenges but could be improved upon with further investigation.

While some Cr foils are commercially available, Cr foils are very difficult to manipulate into discs since the material is brittle. The Cr metal powder was crafted into a pressed pellet for in the production of ^52^Mn. While this target configuration works well and could be consistently used for production of ^52^Mn it cannot be reused or recycled for a cost-effective means to utilize enriched ^52^Cr. ^52^Cr could be exchanged for natural Cr in this target configuration to prevent the production of ^54^Mn if the cost of the enriched material was not a factor. Ideally, the enriched material would be 100% recycled and the target could be reused multiple times which lead to exploration of additional Cr target configurations.

The Cr_2_O_3_ target does have the potential to be recycled since the final Cr species from the purification is CrCl_3_. The CrCl_3_ could potentially be reacted to make more Cr_2_O_3_ to make a new target. However, we were not able to dissolve this material which leads to purification issues. Finally, the electroplated Cr (III) target is the most promising since it has the potential to be recycled, can be dissolved/purified and it is less toxic than the well-known Cr(VI) electrodeposition. In order to improve the yield of Cr(III) on the Au surface new techniques need to be explored with the aim of yields greater than 90% at a thickness greater than 0.2 mm.

The concentration of the eluting solution affected greatly the AMA of the ^52^Mn produced. Higher concentrations of HCl acid selectively released ^52^Mn from the resin bed.

## Conclusion

Enriched targets for the production of ^52^Mn should continue to be investigated in order to obtain a recyclable target that increase the yield and decreases the time of irradiation. Cr foil and Cr metal powder targets are routinely used for the production of ^52^Mn, however, these targets cannot be recycled in order to move to the enriched ^52^Cr target material. Cr_2_O_3_ is not feasible to use for natural or enriched targets because of the dissolution issues. Although the target is capable of giving higher yields when enriched material is used and it is possible to recycle through the CrCl_3_ to Cr_2_O_3_ reaction, it has been a challenge to dissolve and separate the ^52^Mn from the Cr. Finally, the Cr electroplated targets are the most promising for the future of enriched ^52^Cr targets for ^52^Mn production. These targets have the potential to be recycled by using the final CrCl_3_ in the new electroplating solution and the plated Cr can endure more current than the other targets described here leading to increased yields.

## Data Availability

The datasets generated during and/or analyzed during the current study are available from the corresponding author on reasonable request.
